# Small, dense LDL-C as a predictor of cardiovascular risk and benefit of alirocumab in patients with recent acute coronary syndrome receiving optimized statin treatment

**DOI:** 10.1016/j.ajpc.2026.101643

**Published:** 2026-04-17

**Authors:** Gregory G. Schwartz, Michael Szarek, Christa M. Cobbaert, L. Renee Ruhaak, Markus Schwertfeger, Deepak L. Bhatt, Vera A. Bittner, Shaun G. Goodman, Robert A. Harrington, Irena Stevanovic, Hagai Tavori, Esther Reijnders, Fred Romijn, Nicolaas J.M. van Neer, Harvey D. White, J.W. Jukema, P. Gabriel Steg

**Affiliations:** a Division of Cardiology, University of Colorado School of Medicine, Aurora, CO, USA; b CPC Clinical Research, Aurora, CO, USA; c State University of New York Downstate Health Sciences University, Brooklyn, NY, USA; d Department of Clinical Chemistry and Laboratory Medicine, Leiden University Medical Center, Leiden, The Netherlands; e Roche Diagnostics, Zug, Switzerland; f Mount Sinai Fuster Heart Hospital, Icahn School of Medicine, New York, NY, USA; g Division of Cardiovascular Disease, University of Alabama at Birmingham, Birmingham, AL, USA; h Canadian VIGOUR Centre, University of Alberta, Edmonton, Alberta, Canada; i St Michael’s Hospital, University of Toronto, Ontario, Canada; j Weill Cornell Medicine, New York, NY; k Sanofi, Paris, France; l Yakum, Israel; m Green Lane Cardiovascular Research Unit, Te Whatu Ora-Health New Zealand, Te Toka Tumai, and University of Auckland, New Zealand; n Department of Cardiology, Leiden University Medical Center, Leiden, The Netherlands; o Netherlands Heart Institute, Utrecht, the Netherlands; p Université Paris-Cité, INSERM-UMR1148, Assistance Publique-Hôpitaux de Paris, Hôpital Bichat, French Alliance for Cardiovascular Trials, and Institut Universitaire de France, Paris, France

**Keywords:** Small, dense LDL, Acute coronary syndrome, Alirocumab

## Abstract

**Introduction:**

Small, dense low-density lipoprotein (sdLDL) particles are considered a highly atherogenic subfraction of LDL. Automated biochemical measurement of sdLDL cholesterol (sdLDL-C) has good fidelity to gold-standard measurements. We evaluated relationships of sdLDL-C and LDL-C to risk of major adverse cardiovascular events (MACE) and treatment benefit of the PCSK9-directed monoclonal antibody alirocumab in patients with recent acute coronary syndrome (ACS) and elevated atherogenic lipoproteins despite optimized statin treatment.

**Methods:**

Analyses comprised 11,837 participants in the ODYSSEY OUTCOMES trial randomized to receive alirocumab or placebo. sdLDL-C was measured using the Denka method; baseline LDL-C was calculated with the Friedewald formula. In the placebo group (*n* = 5920), cubic splines depicted relationships of sdLDL-C, LDL-C, and their ratio to risk of MACE (cardiovascular death, non-fatal myocardial infarction, ischemic stroke, hospitalization for unstable angina, and ischemia-driven coronary revascularization). Treatment hazard ratio (HR) was calculated across sdLDL-C and LDL-C ranges.

**Results:**

Over 2.8 years median follow-up, risk of MACE in the placebo group increased with higher baseline sdLDL-C or LDL-C with nearly superimposable splines and without variation according to sdLDL-C/LDL-C. Findings were similar in patients with greater or lesser degrees of insulin resistance. Alirocumab reduced risk of MACE (HR 0.87, 95% CI 0.79, 0.95). Treatment HR did not vary significantly across the range of LDL-C or sdLDL-C.

**Conclusion:**

In patients with recent ACS on optimized statin treatment, sdLDL-C and LDL-C similarly predict risk of MACE and benefit of alirocumab treatment. Measurement of sdLDL-C does not appear to provide additional prognostic or predictive information in this population.

## 1 Introduction

Low-density lipoprotein (LDL) particles may be characterized by their size and density. Small, dense LDL particles (sdLDL) are believed to be a highly atherogenic subfraction of LDL due to their prolonged residence time in circulation, propensity for adherence to and penetration of vascular endothelium, and susceptibility to oxidation [1,2]. Gold-standard methods for characterizing lipoprotein particle size and density are nuclear magnetic resonance spectroscopy and gradient ultracentrifugation, techniques that are not readily applicable to large trial cohorts or routine clinical testing. As an alternative, automated biochemical methods have been developed to measure the cholesterol content of sdLDL (sdLDL-C) and demonstrate strong correlation with the gold standard techniques [3].

A higher concentration of sdLDL or sdLDL-C has consistently associated with greater risk of incident coronary heart disease in observational cohorts and in meta-analyses, even with adjustment for covariates including diabetes [[4], [5], [6]]. However, there is a paucity of data with a lack of concordance addressing the question of whether sdLDL or sdLDL-C predicts risk of recurrent major adverse cardiovascular events (MACE) in patients with established atherosclerotic cardiovascular disease. For example, two of the largest analyses to date using biochemical measurement of sdLDL-C have come to different conclusions. In a single-center cohort of 1034 patients with stable coronary artery disease, 59% of whom were statin-treated, sdLDL-C predicted subsequent coronary heart disease events independently of apolipoprotein B or non-high-density lipoprotein cholesterol [7]. In contrast, in the Atherothrombosis Intervention in Metabolic Syndrome with Low HDL/High Triglycerides and Impact on Global Health Outcomes (AIM-HIGH) trial in 3094 simvastatin-treated patients with established atherosclerotic cardiovascular disease, baseline levels of sdLDL-C did not differ between those who did or did not experience MACE (death from coronary heart disease, nonfatal myocardial infarction, ischemic stroke, hospitalization for unstable angina or cerebrovascular revascularization) [8]. Based in part upon these findings, there is no current, strong guidance to assess sdLDL in clinical practice [9].

High-intensity statin therapy reduces LDL-C levels by more than 50% from an untreated baseline. Further addition of inhibitors of proprotein convertase subtilisin/kexin type 9 (PCSK9) results in net reduction of LDL-C levels by more than 75% from an untreated baseline with strong concordance between the reductions in LDL-C and sdLDL-C [10]. In this context, it is uncertain whether sdLDL-C adds prognostic and predictive information to conventional LDL-C in patients with a recent ACS treated with high-intensity statin without or with concurrent PCSK9 inhibitor.

## 2 Methods

To address this research question, we performed a post hoc analysis of the ODYSSEY OUTCOMES trial (clinicaltrials.gov NCT01663402), which compared the PCSK9 monoclonal antibody, alirocumab, with placebo in 18,924 patients with recent ACS and LDL-C ≥ 70 mg/dL, non-high-density lipoprotein cholesterol (non-HDL-C) ≥ 100 mg/dL, or apolipoprotein B ≥ 80 mg/dL despite background treatment with high-intensity or maximum-tolerated statin therapy at 1315 sites in 57 countries [11]. Patients were randomly assigned to treatment with alirocumab 75 mg subcutaneously every 2 weeks, or matching placebo, and followed for a median of 2.8 years. All participants provided written informed consent. The trial was approved by the institutional review board or ethics committee at each site. The present analysis comprised 11,837 trial participants who participated in a biomarker substudy.

Serum samples obtained at baseline (*n* = 11,837) and after 4 months of assigned study treatment (*n* = 10,967) were shipped and maintained in frozen state until analysis of sdLDL-C at Leiden University Medical Center, Leiden, The Netherlands. sdLDL-C was measured using the Roche Diagnostics-Denka (Niigata, Japan) method on a Roche Cobas C502 autoanalyzer [12] with a between-run coefficient of variation of 3.07–3.62% [13]. Baseline LDL-C was calculated with the Friedewald formula from direct measurements of total cholesterol, HDL-C, and triglyceride. In 4-month samples, LDL-C was measured by preparative ultracentrifugation and beta quantitation when the Friedwald calculation was below 15 mg/dL.

For this analysis, the clinical outcome of interest was a 5-part MACE composite comprising cardiovascular death, non-fatal myocardial infarction or ischemic stroke, hospitalization for unstable angina, and ischemia-driven coronary revascularization. Outcome events were blindly adjudicated by a committee of cardiologists and neurologists.

Correlation coefficients (Spearman’s r) between baseline sdLDL-C and LDL-C or apolipoprotein B were calculated. Natural cubic splines depicted the relationships of baseline sdLDL-C, LDL-C, and their ratio to the risk of MACE in the placebo group. In a Cox regression model relating baseline LDL-C to risk of MACE in the placebo group, we determined whether addition of sdLDL-C to the model provided significant incremental information.

Because insulin resistance is associated with smaller, denser LDL particles [1], we determined whether sdLDL-C contributed information specifically in insulin-resistant patients. Fasting insulin and glucose were used to calculate the homeostasis model assessment of insulin resistance (HOMA-IR) [14]. HOMA-IR was dichotomized at its median value and spline relationships of LDL-C and sdLDL-C to MACE were examined in both HOMA-IR subgroups.

Apolipoprotein B, an integrated measure of atherogenic lipoprotein particles, was measured as previously reported [15]. The comparative relationships of sdLDL-C and apolipoprotein B with risk of MACE in the placebo group was examined with natural cubic splines. To further describe the independent prognostic information provided by sdLDL-C or LDL-C versus apoB, splines for the biomarkers were compared for concordance of observed versus predicted outcomes among all possible pairs of participants where one participant has a MACE and the other participant does not.

At month 4 of treatment with alirocumab or placebo, the changes from baseline of sdLDL-C, LDL-C, and their ratio were examined. To determine whether baseline sdLDL-C or LDL-C influenced the clinical benefit of treatment with alirocumab, the treatment hazard ratio (HR) for MACE (alirocumab/placebo) was examined as a function of each variable.

## 3 Results

Table 1 shows baseline characteristics of the analysis cohort including levels of sdLDL-C, LDL-C, and their ratio, as well as levels at month 4 of assigned study treatment. Baseline characteristics were well-balanced between treatment groups and similar to those of the full study cohort [11], except that patients comprising the analysis cohort were less likely enrolled at Central or Eastern European sites and more likely enrolled at North American sites. Notably, 29.4% of the analysis cohort had diabetes, 89.1% were treated with a high-intensity statin regimen, and median baseline apolipoprotein B concentration was 80 mg/dL. Aggregate median baseline levels of sdLDL-C, LDL-C, and their ratio were 30.7 mg/dL, 86.2 mg/dL, and 0.35, respectively. There was moderately strong correlation between baseline sdLDL-C and LDL-C (*r* = 0.55) and between baseline sdLDL-C and apolipoprotein B (*r* = 0.81).

**Table 1:** Characteristics of the analysis cohort.

	Alirocumab (n=5917)	Placebo (n=5920)
Age, years	58 (52, 65)	58 (52, 65)
Sex, female	1406 (23.8)	1419 (24.0)
Race		
White	4736 (80.0)	4779 (80.7)
Asian	717 (12.1)	712 (12.0)
Black	166 (2.8)	156 (2.6)
Other	298 (5.0)	273 (4.6)
Region of enrolment		
Central/Eastern Europe	1149 (19.4)	1140 (19.3)
Western Europe	1397 (23.6)	1406 (23.8)
Canada/USA	1388 (23.5)	1390 (23.5)
Latin America	787 (13.3)	782 (13.2)
Asia	686 (11.6)	683 (11.5)
Rest of World	510 (8.6)	519 (8.8)
Medical history and background lipid-lowering therapy		
Hypertension	3767 (63.7)	3686 (62.3)
Diabetes mellitus	1725 (29.2)	1756 (29.7)
Current tobacco smoker	1421 (24.0)	1431 (24.2)
Coronary revascularization for index ACS	4303 (72.7)	4371 (73.8)
Polyvascular disease*	483 (8.2)	498 (8.4)
High-intensity statin	5241 (88.6)	5307 (89.6)
Low- or moderate-intensity statin	471 (8.0)	422 (7.1)
Ezetimibe	184 (3.1)	212 (3.6)
Laboratory data		
LDL-C, mg/dL	86.2 (72.7, 103.6)	86.2 (73.1, 104.0)
sdLDL-C, mg/dL	30.9 (22.8, 42.5)	30.5 (22.8, 42.9)
sdLDL-C/LDL-C	0.35 (0.27, 0.46)	0.34 (0.27, 0.46)
HDL-C, mg/dL	42.1 (36.0, 49.9)	42.1 (36.0, 49.9)
Triglyceride, mg/dL	130.2 (94.8, 180.6)	131.1 (94.8, 184.2)
ApoB, mg/dL	79 (69, 93)	80 (69, 94)
HbA1c, %	5.8 (5.5, 6.3)	5.8 (5.5, 6.4)
eGFR (mL/min/1.73 m^2^)	77.9 (67.2, 90.1)	78.2 (67.4, 90.4)
HOMA-IR^†^	3.30 (2.02, 5.70)	3.32 (2.05, 5.78)
Changes from baseline to month 4		
LDL-C, mg/dL	-54.9 (-71.1, -38.3)	1.2 (-11.6, 13.9)
sdLDL-C, mg/dL	-16.2 (-25.1, -9.3)	1.2 (-5.4, 8.1)
sdLDL-C/LDL-C	0.08 (0, 0.22)	0.01 (-0.05, 0.07)

Continuous variables are median (Q1, Q3). Dichotomous variables are number (%). *Coronary and cerebrovascular or peripheral artery disease. ^†^For HOMA-IR, *n* = 5696 (placebo) and *n* = 5706 (alirocumab).

**Fig. 1:**
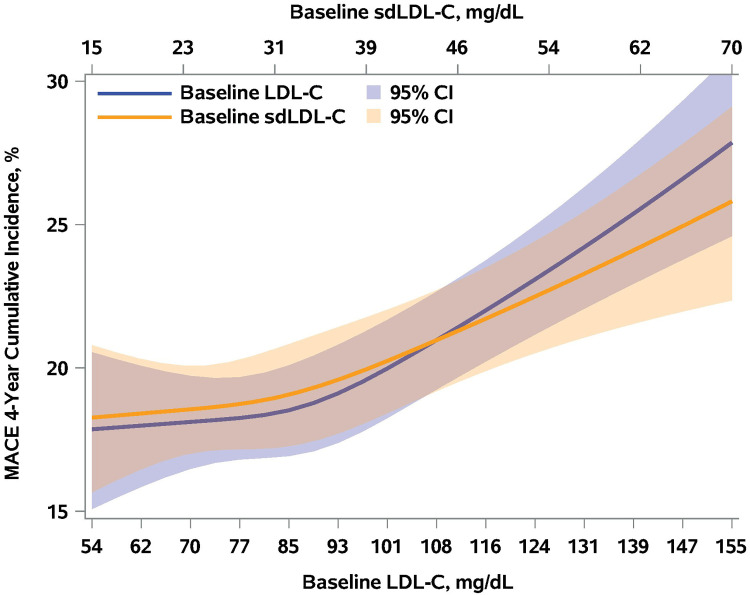
Baseline sdLDL-C and LDL-C versus MACE in the placebo group. Natural cubic splines (plotted over a range of 5th to 95th percentile of each independent variable) depicting: A. Baseline sdLDL-C and LDL-C versus MACE in the placebo group (*n* = 5920). Spline effect *p* = 0.0005 for sdLDL-C and *p* < 0.0001 for LDL-C. CI, confidence interval. HR, hazard ratio. LDL-C, low-density lipoprotein cholesterol. MACE, major adverse cardiovascular events. sdLDL-C, cholesterol content of small, dense, low-density lipoprotein.

**Fig. 2:**
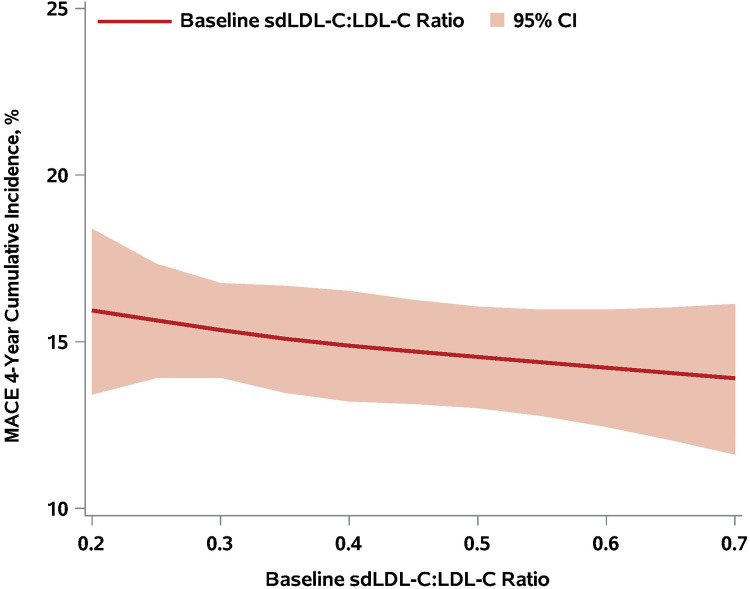
Baseline ratio of sdLDL-C/LDL-C and risk of MACE in the placebo group *N* = 5920. There was no significant relationship (spline effect *p* = 0.44). See Fig. 1 for abbreviations.

In the placebo group (*n* = 5920), the risk of MACE increased monotonically with concentrations of baseline sdLDL-C and LDL-C with nearly superimposable splines (Fig. 1). The risk of MACE in the placebo group did not vary with the baseline sdLDL-C/LDL-C ratio (Fig. 2; spline *p* = 0.44). Cox regression showed that a 10 mg/dL higher baseline LDL-C level in the placebo group was associated with a HR for MACE of 1.059 (95% CI, 1.040, 1.080), *p* < 0.0001. With addition of sdLDL-C to the model, LDL-C remained associated with MACE (HR 1.052 [95% CI, 1.028, 1.077], *p* < 0.0001 per 10 mg/dL higher baseline level), whereas sdLDL-C did not maintain a significant relationship with MACE (HR 1.024 [95% CI, 0.979, 1.070], *p* = 0.31 per 10 mg/dL higher baseline level). Thus, findings from regression and spline analyses were congruent.

**Fig. 3:**
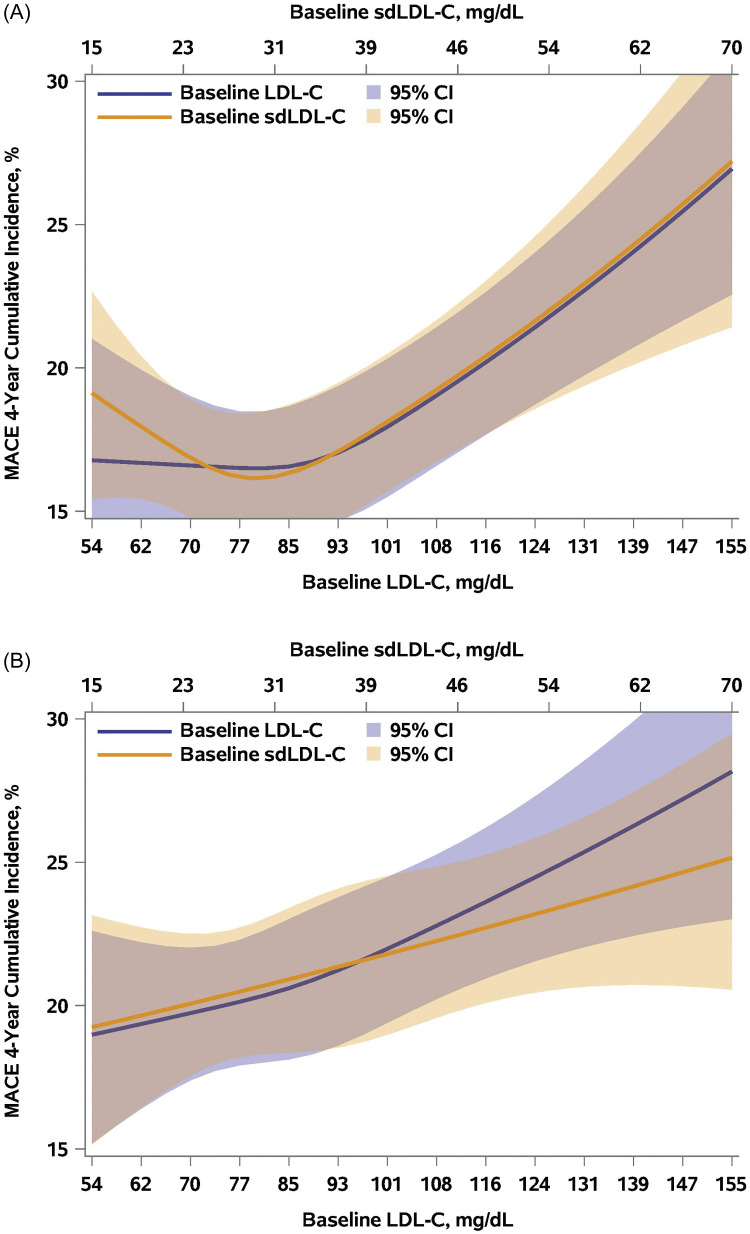
A. Baseline sdLDL-C and LDL-C versus MACE in the placebo group with HOMA-IR below the median value of 3.31 (*n* = 2839). B. Baseline sdLDL-C and LDL-C versus MACE in the placebo group with HOMA-IR at or above the median value of 3.31 (*n* = 2858). HOMA-IR, homeostasis model assessment of insulin resistance. See Fig. 1 for other abbreviations.

At baseline, median HOMA-IR was 3.31. In subgroups with HOMA-IR below versus at or above this level, median (Q1, Q3) HOMA-IR was 2.04 (1.45, 2.62) versus 5.74 (4.24, 9.22), sdLDL-C was 28.0 (21.2, 38.6) versus 33.9 (24.9, 46.1) mg/dL, and LDL-C was 87.0 (74.1, 104.6) versus 85.3 (71.0, 103.1) mg/dL, respectively. Thus, both the absolute concentration of sdLDL-C and its proportion of total LDL-C were higher in patients with a greater degree of insulin resistance. Nonetheless, splines relating sdLDL-C and LDL-C to risk of MACE remained largely superimposable in both HOMA-IR categories (Fig. 3).

**Fig. 4:**
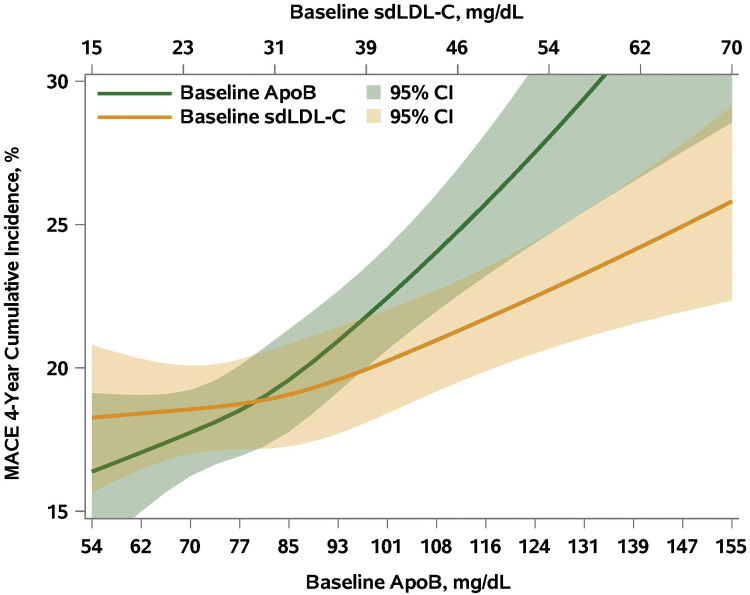
Baseline sdLDL-C and ApoB versus MACE in the placebo group (*n* = 5920). ApoB, apolipoprotein B. See Fig. 1 for other abbreviations.

Fig. 4 compares splines depicting the risk of MACE versus apolipoprotein B or sdLDL-C. Concordance (95% CI) was 53.4% (51.1%, 55.6%) for sdLDL-C, which was significantly lower than the 55.3% (53.0%, 57.6%) for apolipoprotein B (*p* = 0.0127), indicating that apolipoprotein B was a better predictor of MACE than sdLDL-C. In contrast, the concordance of LDL-C [55.2% (52.8%, 57.6%)] was nearly identical to that of apolipoprotein B, indicating that those two biomarkers predicted MACE similarly (*p* = 0.83).

**Fig. 5:**
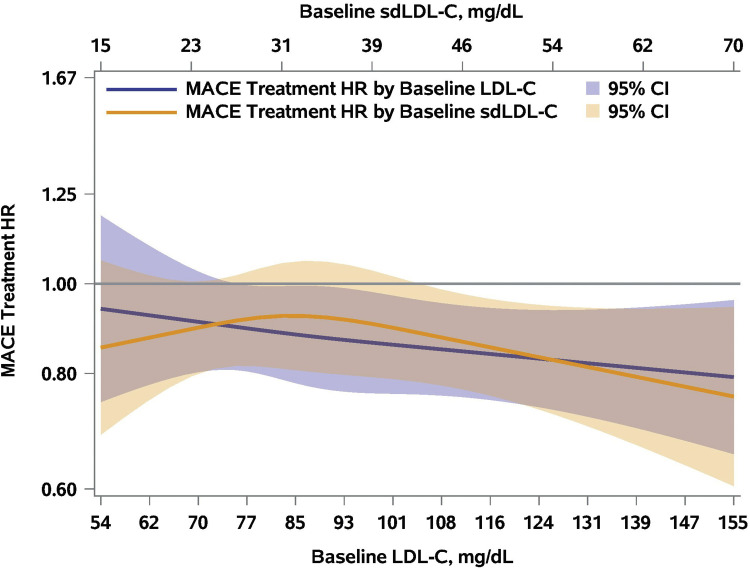
Treatment hazard ratio for MACE by baseline sdLDL-C and LDL-C (*n* = 5917 and *n* = 5920 for the alirocumab and placebo groups, respectively). The treatment hazard ratio did not vary significantly with either predictor variable (spline effect *p* = 0.36 for sdLDL-C and *p* = 0.50 for LDL-C). See Fig. 1 for abbreviations.

At month 4, alirocumab reduced sdLDL-C and LDL-C by median 56.3% and 65.2%, respectively, without significant change in their ratio (**Table**). Overall, alirocumab reduced the risk of MACE compared with placebo (HR 0.87, 95% CI 0.79, 0.95). However, the treatment HR did not vary across the range of either sdLDL-C or LDL-C (Fig. 5; spline p-values 0.36 and 0.50, respectively).

## 4 Discussion

There are several key findings from this analysis, summarized in the **Central Illustration**. First, in patients with recent acute coronary syndrome and LDL-C ≥ 70 mg/dL on optimized statin treatment, sdLDL-C and LDL-C are equivalent predictors of the risk of MACE. Second, findings were similar when patients were dichotomized according to median HOMA-IR, thus indicating that sdLDL-C does not provide superior prognostic information to LDL-C even among patients with marked insulin resistance in whom sdLDL-C concentration was elevated. Third, the treatment hazard ratio for MACE (alirocumab/placebo) remained relatively constant across the ranges of sdLDL-C and LDL-C, indicating that the benefit of alirocumab treatment does not depend on the levels of either measure. Finally, apolipoprotein B, an integrated measure of all atherogenic particles, was a stronger predictor of the risk of MACE than sdLDL-C.

**Figure:**
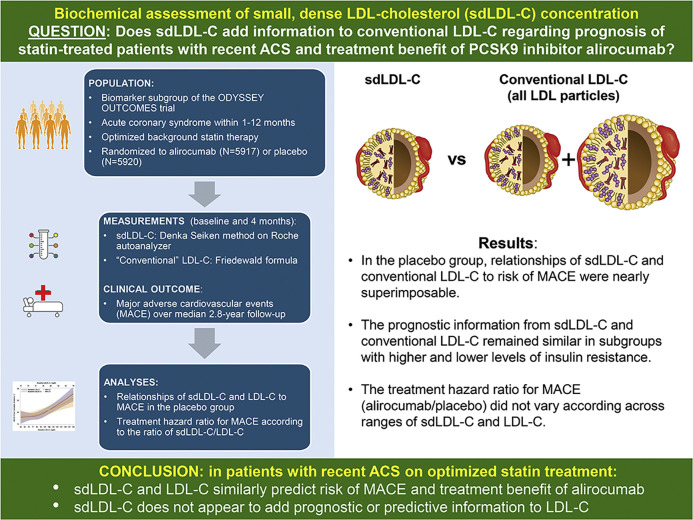
**Central Illustration**. Small, dense LDL-C as a predictor of cardiovascular risk and benefit of alirocumab treatment in patients with recent acute coronary syndrome receiving optimized statin treatment.

Similar prediction of risk of MACE by sdLDL-C or LDL-C may reflect the fact that all LDL particles contain a single apolipoprotein B moiety, and the total number of apolipoprotein B-containing particles may be an overriding determinant of lipoprotein-attributable cardiovascular risk [16]. Even among patients with marked insulin resistance (median HOMA-IR 5.74) and elevated sdLDL-C levels there was no apparent advantage of sdLDL-C over LDL-C in prediction of MACE. Moreover, higher sdLDL-C levels were not associated with greater relative reduction in the risk of MACE with alirocumab. In sum, biochemical measurement of sdLDL-C does not appear to provide additional prognostic information to LDL-C in statin-treated patients with recent ACS or to help guide treatment with a PCSK9 inhibitor in such patients.

Strengths of the current analysis include the largest dataset to date relating levels of sdLDL-C to risk of MACE in a secondary prevention cohort, and the only analysis to date assessing whether the cardiovascular benefit of PCSK9 inhibition depends upon sdLDL-C levels. Among limitations, our analysis was performed post hoc in a subset (62.6%) of the overall ODYSSEY OUTCOMES cohort with available samples. To increase the number of events and analytic power, we used an expanded 5-part MACE endpoint including ischemia-driven coronary revascularization rather than the trial primary outcome measure, a 4-part MACE composite without coronary revascularization. For these reasons, the analysis must be considered exploratory. However, there were no qualitative differences in findings using the primary outcome measure of the trial (data not shown). Baseline LDL-C was calculated with the Friedewald formula, but any resulting inaccuracies were mitigated by the trial’s exclusion of patients with triglyceride levels above 400 mg/dL or with low LDL-C. The relationship of biochemical sdLDL-C with gold-standard ultracentrifugation or NMR spectroscopy is good, but imperfect [3,17]. It is uncertain whether use of gold-standard methods would have affected the findings. Prospective assessment of sdLDL-C in future trials of new lipid-lowering therapies may provide further insight into the potential clinical utility of this biomarker.

## Funding

The ODYSSEY OUTCOMES trial was funded by Sanofi and Regeneron Pharmaceuticals. Measurement of sdLDL-C was supported by 10.13039/100016545Roche Diagnostics. The authors appreciate assistance in preparing the Central Illustration from MedLink Healthcare Communications, London, U.K. (funded by Sanofi).

## CRediT authorship contribution statement

**Gregory G. Schwartz:** Writing – original draft, Investigation, Funding acquisition, Conceptualization. **Michael Szarek:** Formal analysis, Data curation. **Christa M. Cobbaert:** Writing – review & editing, Validation, Supervision, Project administration, Methodology, Investigation. **L. Renee Ruhaak:** Validation, Methodology, Investigation. **Markus Schwertfeger:** Writing – review & editing, Resources, Funding acquisition. **Deepak L. Bhatt:** Writing – review & editing, Investigation. **Vera A. Bittner:** Writing – review & editing, Investigation. **Shaun G. Goodman:** Writing – review & editing, Investigation. **Robert A. Harrington:** Writing – review & editing, Investigation. **Irena Stevanovic:** Writing – review & editing. **Hagai Tavori:** Writing – review & editing. **Esther Reijnders:** Writing – review & editing, Validation, Investigation. **Fred Romijn:** Writing – review & editing, Validation, Investigation. **Nicolaas J.M. van Neer:** Writing – review & editing. **Harvey D. White:** Writing – review & editing, Investigation. **J.W. Jukema:** Writing – review & editing, Supervision, Project administration, Investigation, Funding acquisition. **P. Gabriel Steg:** Writing – review & editing, Supervision, Investigation, Funding acquisition.

## Declaration of competing interest

The authors declare the following financial interests/personal relationships which may be considered as potential competing interests:

**Gregory Schwartz** reports research support from the US Department of Veterans Affairs, research support to the University of Colorado from 10.13039/100004325AstraZeneca, 10.13039/100004339Sanofi and 10.13039/501100022030Silence Therapeutics, and support for travel to trial meetings from the University of Oxford. **Michael Szarek** reports receives salary support from CPC Clinical Research, a non-profit academic research organization affiliated with the University of Colorado, that receives or has received research grant/consulting funding between July 2021 and July 2025 from the following organizations: 35Pharma, 10.13039/100001316Abbott Laboratories, Agios Pharmaceuticals, Alexion Pharma Godo Kaisha, 10.13039/100000968American Heart Association, American Journal of Managed Care, Amgen USA, Anthos Therapeutics, 10.13039/100014931Arrowhead Pharmaceuticals, AstraZeneca, Autonomy Bio, 10.13039/100004326Bayer, Bayer Aktiengesellschaft, 10.13039/100005615Beth Israel Deaconess Medical Center, Better Therapeutics, Boston Clinical Research Institute, 10.13039/100002491Bristol-Myers Squibb, Cleerly, Clergy United for the Transformation of Sandtown, Colorado Dept of Public Health and Environment, Congress Inc, Cook Regentec, Eidos Therapeutics, EluraBio, Esperion Therapeutics, Faraday Pharmaceuticals, Gasherbrum Bio, 10.13039/100020483Insmed, IsomAb, JanOne Biotech Holdings, Janssen Global Services, Janssen Pharmaceuticals, Janssen Scientific Affairs, Las Animas and Huerfano Counties District Health Dept, Lexicon Pharmaceuticals, Lilly USA, Medison Pharma, 10.13039/100007050Medpace, Merck Sharp & Dohme, Nectero Medical, NewAmsterdam Pharma, 10.13039/100008272Novartis Pharmaceuticals, Novo Nordisk, 10.13039/100004319Pfizer, Piper Sandler & Co, PPD Development, Prothena Biosciences, 10.13039/100009857Regeneron, Regents of the University of Colorado, Sanifit Therapeutics, 10.13039/100004339Sanofi, Silence Therapeutics, 10.13039/100023812Stanford University, Stealth BioTherapeutics, The Brigham and Women's Hospital, Thrombosis Research Institute, Tourmaline Bio, 10.13039/100007494University of Colorado, 10.13039/100008603University of Pittsburgh, VarmX, Verve Therapeutics, WraSer, and reports serving as a consultant or research support (or both) from 10.13039/100014389Amarin, Lexicon, NewAmsterdam Pharma, 10.13039/100004336Novartis, 10.13039/100009857Regeneron, 10.13039/100004339Sanofi, 10.13039/501100022030Silence, and Tourmaline. **Christa Cobbaert** reports a research collaboration with 10.13039/100012288Roche as well as research grants from 10.13039/100016545Roche Diagnostics. **L. Renee Ruhaak** has no disclosures. **Markus Schwertfeger** is an employee of Roche and may hold shares in the company. **Deepak L. Bhatt** discloses the following relationships - Advisory Board: Angiowave, Antlia Bioscience, Bayer, Boehringer Ingelheim, CellProthera, Cereno Scientific, E-Star Biotech, High Enroll, Janssen, Level Ex, McKinsey, Medscape Cardiology, Merck, NirvaMed, Novo Nordisk, Repair Biotechnologies, Stasys, Tourmaline Bio; Board of Directors: American Heart Association New York City, Angiowave (stock options), Bristol Myers Squibb (stock), DRS.LINQ (stock options), High Enroll (stock); Consultant: Alnylam, Altimmune, Broadview Ventures, Corcept Therapeutics, Corsera, GlaxoSmithKline, Hims, SERB, SFJ, Summa Therapeutics, Worldwide Clinical Trials; Data Monitoring Committees: Acesion Pharma, Assistance Publique-Hôpitaux de Paris, Baim Institute for Clinical Research, Boston Scientific (Chair, PEITHO trial), Cleveland Clinic, Contego Medical (Chair, PERFORMANCE 2), Duke Clinical Research Institute, Mayo Clinic, Mount Sinai School of Medicine (for the ABILITY-DM trial, funded by 10.13039/100030895Concept Medical; for ALLAY-HF, funded by Alleviant Medical), 10.13039/100004336Novartis, 10.13039/100030936Population Health Research Institute; 10.13039/100011132Rutgers University (for the 10.13039/100000052NIH-funded MINT Trial); Honoraria: 10.13039/100005485American College of Cardiology (Senior Associate Editor, Clinical Trials and News, ACC.org; Chair, ACC Accreditation Oversight Committee), Arnold and Porter law firm (work related to Sanofi/Bristol-Myers Squibb clopidogrel litigation), Baim Institute for Clinical Research (AEGIS-II executive committee funded by 10.13039/100008322CSL Behring), Belvoir Publications (Editor in Chief, Harvard Heart Letter), Canadian Medical and Surgical Knowledge Translation Research Group (clinical trial steering committees), 10.13039/100008322CSL Behring (AHA lecture), 10.13039/100006513Duke Clinical Research Institute, Engage Health Media, HMP Global (Editor in Chief, Journal of Invasive Cardiology), Medtelligence/ReachMD (CME steering committees), MJH Life Sciences, Oakstone CME (Course Director, Comprehensive Review of Interventional Cardiology), 10.13039/100004320Philips (Becker's Webinar on AI), 10.13039/100030936Population Health Research Institute, WebMD (CME steering committees), 10.13039/100005134Wiley (steering committee); Other: Clinical Cardiology (Deputy Editor); Progress in Cardiovascular Diseases (Deputy Editor); Patent: Sotagliflozin (named on a patent for sotagliflozin assigned to Brigham and Women's Hospital who assigned to Lexicon; neither I nor Brigham and Women's Hospital receive any income from this patent); Research Funding: 10.13039/100000046Abbott, Acesion Pharma, Afimmune, Alnylam, Amarin, 10.13039/100002429Amgen, 10.13039/100004325AstraZeneca, 10.13039/100007330Atricure, 10.13039/100004326Bayer, 10.13039/100001003Boehringer Ingelheim, 10.13039/100016242Boston Scientific, CellProthera, Cereno Scientific, 10.13039/100020218Chiesi, Cleerly, 10.13039/100008322CSL Behring, Faraday Pharmaceuticals, Fractyl, 10.13039/501100016198Idorsia, 10.13039/501100024879Janssen, 10.13039/501100003490Javelin, Lexicon, 10.13039/100021235Lilly, 10.13039/100004374Medtronic, 10.13039/100004334Merck, MiRUS, Moderna, 10.13039/100004336Novartis, 10.13039/501100004191Novo Nordisk, 10.13039/100004319Pfizer, PhaseBio, 10.13039/100009857Regeneron, Reid Hoffman Foundation, 10.13039/100031177Roche, 10.13039/100004339Sanofi, Stasys, 10.13039/10002088789Bio; Royalties: Elsevier (Editor, Braunwald’s Heart Disease); Site Co-Investigator: Cleerly. **Vera A. Bittner** reports grant support from 10.13039/100004339Sanofi, 10.13039/100009857Regeneron Pharmaceuticals, 10.13039/100002429Amgen, AstraZeneca, 10.13039/501100016186DalCor, 10.13039/501100022336Esperion, and 10.13039/100004336Novartis; consulting fees from 10.13039/100004319Pfizer; honoraria from Medscape; and fees for data safety monitoring boards for the National Institutes of Health and for Verve Therapeutics. **Shaun G. Goodman** reports research grant support (e.g., steering committee or data and safety monitoring committee) or speaker/consulting honoraria (e.g., advisory boards) from 10.13039/100002429Amgen, 10.13039/100020132Anthos Therapeutics, 10.13039/100004325AstraZeneca, 10.13039/100004326Bayer, 10.13039/100001003Boehringer Ingelheim, 10.13039/100001009Bristol Myers Squibb, 10.13039/100008322CSL Behring, Daiichi-Sankyo/American Regent, 10.13039/100004312Eli Lilly, 10.13039/501100022336Esperion, 10.13039/501100004914Ferring Pharmaceuticals, 10.13039/501100018679HLS Therapeutics, JAMP Pharma, 10.13039/100004334Merck, 10.13039/100012890Novartis, Novo Nordisk A/C, Pendopharm/Pharmascience, 10.13039/100004319Pfizer, 10.13039/100009857Regeneron, 10.13039/100004339Sanofi, 10.13039/501100011725Servier, and Valeo Pharma; and salary support/honoraria from the Heart and Stroke Foundation of Ontario/University of Toronto (Polo) chair, Canadian Heart Research Centre and MD Primer, Canadian VIGOUR Centre, Cleveland Clinic Coordinating Centre for Clinical Research, 10.13039/100006513Duke Clinical Research Institute, New York University Clinical Coordinating Centre, PERFUSE Research Institute, and the TIMI Study Group (Brigham Health). **Robert A. Harrington** reports research relationships with Baim Institute (DSMB), Colorado Prevention Center (CPC) Clinical Research (DSMB), Edwards Lifesciences (RCT), Janssen (RCT), Merck/BWH (DSMB); Consulting relationships: Atropos Health, Basking Biosciences, Bitterroot Bio, BridgeBio Pharma, Corsera Health, Element Science, HeartBeam, Medscape/WebMD; Board of Directors: American Heart Association, Cytokinetics, Marea Therapeutics. **Irena Stevanovic** and **Hagai Tavori** are employees of Sanofi. **Esther Reijnders, Fred Romjin,** and **Nicolaas Van Neer** have no disclosures. **Harvey D. White** reports grant support paid to the institution for the ODYSSEY OUTCOMES trial from Sanofi and Regeneron Pharmaceuticals for the STRENGTH Trial from Omthera Pharmaceuticals for the HEART-FID Study from 10.13039/100016473American Regent, for the Dal GenE study from DalCor Pharma, for the AEGIS II Study from 10.13039/100008322CSL Behring, for the Clear Outcomes Study from 10.13039/501100022336Esperion Therapeutics, for the SOLIST-WHF and SCORED studies from Sanofi Aventis Australia, for the Librexia AF and ACS studies from 10.13039/100008897Janssen, and for ISCHEMIA and the MINT studies from the 10.13039/100000002National Institutes of Health. He also received personal fees as a steering committee member for DalCor Pharma, 10.13039/100008322CSL Behring, Sanofi Australia, 10.13039/100014554Janssen and 10.13039/501100022336Esperion Therapeutics. He was on advisory boards for CSL Behring and Genentech. **J. Wouter Jukema** reports his department has received research grants from and/or was speaker (with or without lecture fees) on a.o. (CME accredited) meetings sponsored/supported by 10.13039/100014386Abbott, 10.13039/100014389Amarin, 10.13039/100002429Amgen, Athera, 10.13039/501100005035Biotronik, 10.13039/100016242Boston Scientific, 10.13039/501100016186Dalcor, 10.13039/501100022274Daiichi Sankyo, 10.13039/100006520Edwards Lifesciences, GE Healthcare, Johnson and 10.13039/100004331Johnson, 10.13039/501100014062Lilly, 10.13039/100016304Medtronic, Merck-Schering-Plough, 10.13039/100004336Novartis, 10.13039/501100004191Novo Nordisk, 10.13039/501100000948Pfizer, 10.13039/100026405Roche, Sanofi Aventis, Shockwave Medical, the Netherlands Heart Foundation, CardioVascular Research the Netherlands (CVON), the Netherlands Heart Institute and the European Community Framework KP7 Program. **Harvey D. White** reports grant support paid to the institution for the ODYSSEY OUTCOMES trial from 10.13039/100004339Sanofi and 10.13039/100009857Regeneron Pharmaceuticals, for the STRENGTH Trial from Omthera Pharmaceuticals for the HEART-FID Study from 10.13039/100016473American Regent, for the Dal GenE study from DalCor Pharma, for the AEGIS II Study from 10.13039/501100024776CSL Behring, for the Clear Outcomes Study from 10.13039/501100022336Esperion Therapeutics, for the SOLIST-WHF and SCORED studies from Sanofi Aventis Australia, for the Librexia AF and ACS studies from 10.13039/501100024879Janssen, and for ISCHEMIA and the MINT studies from the 10.13039/100000002National Institutes of Health. He also received personal fees as a steering committee member for DalCor Pharma, 10.13039/100008322CSL Behring, Sanofi Australia, 10.13039/100008897Janssen and 10.13039/501100022336Esperion Therapeutics. He was on advisory boards for CSL Behring and Genentech. **P. Gabriel Steg** reports grants, personal fees, and nonfinancial support from 10.13039/100004339Sanofi; grants and personal fees from 10.13039/100014389Amarin, 10.13039/501100011725Servier, and 10.13039/100004326Bayer; and personal fees from 10.13039/100002429Amgen, 10.13039/100004325AstraZeneca, 10.13039/501100002750BMS, 10.13039/100008349Boehringer Ingelheim, 10.13039/501100016198Idorsia, 10.13039/100004319Pfizer, and 10.13039/100004336Novartis.
